# Can an Animation Improve Parents' Knowledge and How Does It Compare to Written Information? Development and Survey Evaluation of an Animation for Parents About Prenatal Sequencing

**DOI:** 10.1002/pd.6792

**Published:** 2025-04-02

**Authors:** Morgan Daniel, Hannah McInnes‐Dean, Wing Han Wu, Jane Fisher, Caroline Lafarge, Kerry Leeson‐Beevers, Celine Lewis, Sophie Peet, Dagmar Tapon, Sarah L. Wynn, Lyn S. Chitty, Melissa Hill, Michelle Peter

**Affiliations:** ^1^ North Thames Genomic Laboratory Hub Great Ormond Street Hospital for Children NHS Foundation Trust London UK; ^2^ Genetics and Genomic Medicine UCL Great Ormond Street Institute of Child Health London UK; ^3^ Antenatal Results and Choices London UK; ^4^ The Graduate School University of West London London UK; ^5^ Alström Syndrome UK Torquay UK; ^6^ Population, Policy and Practice UCL Great Ormond Street Institute of Child Health London UK; ^7^ Genetic Alliance UK London UK; ^8^ Queen Charlotte's & Chelsea Hospital Imperial College Healthcare NHS Trust London UK; ^9^ Unique ‐ Rare Chromosome Disorder Support Group Oxted UK

**Keywords:** animation, patient education, patient knowledge, prenatal sequencing

## Abstract

**Objective:**

To develop and evaluate an animation for parents about prenatal sequencing.

**Methods:**

A total of 428 participants who had been pregnant, or whose partner had been pregnant, in the past 24 months. Parents, patient organisation representatives and clinicians co‐designed the animation describing prenatal sequencing (pS). Participants were semi‐randomly assigned to receive one of three interventions (leaflet, animation or both) and answered questions assessing their self‐perceived and objective knowledge before (T1) and after the intervention (T2). Satisfaction with and ease of understanding of the information was assessed at T2.

**Results:**

Survey respondents' (leaflet [*n* = 130], animation [*n* = 153] and both leaflet and animation [*n* = 145]) self‐perceived understanding and knowledge of genetics and objective knowledge of pS increased after all interventions. The leaflet and animation were equally effective in improving the objective knowledge of pS (*F*(2, 421) = 2.48, *p* = 0.085). The animation was viewed positively. Preferences for information format were mixed.

**Conclusion:**

Animated and written information can improve knowledge and understanding of pS. Our animation expands the available information resources for parents offered pS. Further research should evaluate the utility of the animation in a clinical setting.

**Summary**

- What is already known about this topic?
  ◦ Current resources available to parents on prenatal sequencing (pS) are often presented in written format and aimed at academic audiences.
  ◦ Information about pS presented in a way that is accessible to parents is needed.
  ◦ Animations can improve knowledge and satisfaction of understanding genomic sequencing tests.
- What does this study add?
  ◦ Both animated and written information can improve the understanding of pS.
  ◦ Animation is useful for supporting the understanding of pS alongside currently available written resources.

## 1 Introduction

Prenatal sequencing (pS) is known to increase the likelihood of a genetic diagnosis, where structural anomalies identified by prenatal imaging are considered likely to have a genetic aetiology [1]. pS was introduced into routine care in England's National Health Service (NHS) Genomic Medicine Service (GMS) in 2020 [2] and can provide information regarding pregnancy management, prognosis and recurrence risks for future pregnancies [3]. However, it is a complex test offered to parents at an anxious and time‐pressured point in pregnancy [4, 5, 6, 7, 8]. There are multiple considerations when deciding whether or not to have pS, including that it may not provide an explanation for the scan findings or could detect a variant of uncertain significance (VUS). In addition, as pS is ideally performed as trio sequencing (parents and fetus), information about the parents' health and non‐paternity could be identified [3, 9].

In England, parents offered pS are counselled by a healthcare professional who discusses the test procedure, range of possible results, limitations and wider implications. Although written information is available to complement these discussions, parents report wanting information in a range of formats and frequently seek further information online [6]. However, searches for relevant pS terms, most commonly identify academic journal articles, which often require institutional credentials to access resources making it inaccessible to the majority of parents, and commonly use complex and scientific language beyond the average reading age in the United Kingdom [10].

Recent systematic reviews suggest that audiovisual learning aids, such as animations, can be used to convey complex information and increase patient knowledge in a range of health and clinical contexts [11, 12]. In addition, animations, were shown to be effective in improving knowledge and self‐reported understanding of genomic sequencing tests [13, 14] and can improve knowledge of aneuploidy testing compared to information delivered during a standard clinic appointment [15, 16, 17]. Evidence also suggests higher satisfaction when information about genomics is delivered as an animation compared to written information [13, 14].

There is a clear need for pS information to be presented in a way that is accessible to parents for use alongside clinical discussions. Thus, in line with the NHS GMS's vision to deliver an equitable genomics service, we developed and evaluated an animation for parents describing pS that compliments the current resources available through the NHS. To our knowledge, this is the only informative animation aimed at parents that describes pS in the English context. This work was undertaken as part of the Optimising EXome PREnatal Sequencing Services (EXPRESS) study [18]. Our evaluation had three aims: (1) to determine if the animation improves parents' knowledge and understanding of pS, (2) to compare the effectiveness of the animation against a written leaflet with the same information and (3) to determine satisfaction with and perceived value of both the animation and the leaflet.

## 2 Methods

### 2.1 Development of the Animation

The pS animation was developed with input from the EXPRESS patient and public involvement and engagement (PPIE) advisory group, parents offered testing in pregnancy, and clinicians with expertise in pS. The animation content was informed by (a) parent information leaflets and consent forms used in the NHS GMS, (b) guidance on pre‐test counselling content for pS [3, 9, 19], (c) the DISCERN Genetics tool [20] and (d) findings from qualitative interviews with parents offered pS [6]. Animation development involved substantial iterative feedback at multiple points from the PPIE advisory group, parents, and clinicians who contributed to the animation's content, use of language and appearance. The animation describes genome sequencing, what the pS test involves and its possible results and implications. The animation can be viewed on the Antenatal Results and Choices (ARC) website (arc‐uk.org). See Supporting Information S1 for a detailed description of development.

### 2.2 Study Design

An anonymous online survey was conducted that used a pre‐ and post‐intervention assessment.

### 2.3 Participants and Procedure

Participants aged 18 and over who had been pregnant (or had a partner who had been pregnant) in the past 24 months were recruited by a market research company—Dynata (dynata.com). A timeframe of 24 months was considered appropriate for participants to be able to accurately recall their pregnancy and prenatal testing experience. Recruitment targets were set to include a range of ethnic, gender and educational backgrounds. The survey was hosted on the online platform REDcap (project‐redcap.org). An invitation to complete the survey was sent out by Dynata. People interested in taking part clicked a link to redirect them to the survey, where they consented to take part and answered mandatory questions to check eligibility. Eligible participants were then allocated to receive one of three interventions (leaflet, animation or both leaflet and animation). The leaflet was two pages (A4) and the animation was 3 min 49 s long. The content of the leaflet was designed to match that of the animation. Allocation was semi‐random, with participants stratified by ethnicity, gender and education level to ensure balance across the interventions. Participants completed survey questions at two time‐points: T1—before the intervention and T2—immediately after the intervention. Participants who completed the survey received a £2 payment from Dynata. The survey opened for 6 weeks from the 9 February 2024.

### 2.4 Sample Size

An a priori power calculation using G*Power (version 3.1.9.7) [21] indicated 270 participants were required to achieve a medium effect size for subgroup comparisons.

### 2.5 Survey Development and Content

The survey was developed by the researchers with input on content and wording from clinicians and the PPIE advisory group (Supporting Information S1). Participants completed the T1 questions, received the intervention and then completed the T2 questions. T1 questions assessed (1) demographic information, (2) self‐perceived knowledge and understanding of genetics and (3) objective knowledge of pS. T2 questions assessed (1) self‐perceived knowledge and understanding of genetics, (2) objective knowledge of pS and (3) satisfaction and ease of understanding of the animation and/or leaflet. Open‐text boxes allowed further feedback.

### 2.6 Outcome Measures

As prenatal sequencing was only offered in clinical settings in England from October 2020, we were unable to identify an existing tool that specifically addressed our area of interest or measured participants' objective knowledge about this test. For this reason, we designed a survey incorporating questions informed by previous research, the details of which are detailed below.

#### 2.6.1 Self‐Perceived Understanding and Knowledge of Genetics

Three items informed by previous research [14, 22] assessed self‐rated understanding of genetics, understanding of genetic terms and knowledge of genetics terms.

#### 2.6.2 Objective Knowledge

Twelve statements assessed participants' objective knowledge about pS. Respondents could indicate true, false or do not know. Ten statements were informed by existing scales that test knowledge of genomics [23, 24, 25] and two statements were developed by the research team to test concepts specific to pS in the NHS GMS.

#### 2.6.3 Satisfaction and Understanding

Ten items informed by previous research [14, 22] explored participants' satisfaction with and ease of understanding the interventions.

### 2.7 Data Analysis

Descriptive statistics using frequencies and proportions were calculated. Correlations and comparative analyses using ANOVA were conducted to identify differences and any relationships between relevant demographic variables at T1, relationships between outcome variables at T1, and to detect changes between T1 and T2. Spearman's correlation was used to test the association between self‐perceived knowledge and objective knowledge at T1. *Z* tests of proportions were used to assess group differences in self‐perceived knowledge between T1 and T2, and Wilcoxon signed rank tests assessed differences in objective knowledge between T1 and T2. ANCOVA and model comparisons were used to assess the interaction between intervention type and the difference in objective knowledge between T1 and T2. All analyses of quantitative data were conducted using R 4.1.3 [26]. Open‐text comments about the animation and the leaflet allowed participants to describe their thoughts and feelings towards the animation in further detail. The comments in the survey offered valuable supplementary insights that enriched the quantitative data. To systematically manage these qualitative data, MD and WHW used Microsoft Excel to group responses that converged on similar topics. These findings are integrated where relevant to support the quantitative findings.

### 2.8 Missing Data

Minimum times for adequate survey completion were decided in consultation with Dynata, who are experienced in decisions of this nature. Decisions on minimum time to complete the survey were based on the actual length of the animation, the time required to read the leaflet, in addition to the time that it would take to answer the survey questions. Survey responses completed within less than 5 min (leaflet), 6 min (animation) or 7 min (leaflet plus animation) were excluded as these times were considered too short to allow adequate engagement with the intervention and completion of the survey. Of the survey responses excluded for these reasons, the median survey completion time was 3 min 33 s (leaflet), 3 min 36 s (animation) and 4 min 14 s (leaflet plus animation).

## 3 Results

Of 887 respondents who clicked the link to the survey, 218 (24.6%) did not start the survey, 241 (27.2%) were excluded as the time spent completing the survey was below the cut‐off point for their intervention and 428 (48.3%) completed the survey. The median survey completion times were 8 min 45 s (leaflet), 10 min 34 s (animation) and 11 min 43 s (leaflet plus animation). The characteristics of the survey respondents and their genetic testing history are presented in Table 1 and Table S1, Supporting Information S1.

**TABLE 1:** Survey participant characteristics.

	*N* (%)		*N* (%)
	*n* = 428		*n* = 428
Gender		Religion	
Female	234 (55)	Christian	150 (73)
Male	192 (45)	Muslim	44 (21)
Prefer not to say	2 (0)	Hindu	6 (3)
Age, years		Sikh	4 (2)
Mean (SD), range	33.5 (6.5), 18–60	Buddhist	1 (0)
Education		Atheist	1 (0)
Degree and above	253 (64)	Invasive testing in pregnancy?	
Vocational	50 (13)	Yes	92 (22)
A‐level	49 (12)	No	297 (70)
GCSE	45 (11)	Don't know	34 (8)
Ethnicity		Prefer not to say	2 (0)
White/White British	170 (40)	Had a baby identified with a genetic condition in pregnancy?	
Asian/Asian British	89 (21)	Yes	60 (14)
Black/Black British	150 (35)	No	350 (83)
Mixed ethnicity	10 (2)	Don't know	9 (2)
Other ethnicity	2 (0)	Prefer not to say	4 (1)
Prefer not to say	4 (1)	Family member affected by a genetic condition?	
Language		Yes	78 (18)
English	350 (96)	No	315 (74)
Other	36 (4)	Don't know	20 (5)
Number of children		Prefer not to say	4 (1)
Median, IQR	2, 1–2		
Religiosity			
Yes	242 (63)		
No	142 (37)		

*Note:* Some categories do not reflect the total number of respondents since provision of this information was optional; percentages are calculated over known information.

Abbreviations: IQR = interquartile range; SD = standard deviation.

### 3.1 Self‐Perceived Knowledge at T1

At T1, most respondents (70%; *n* = 297) felt they had ‘Some’ understanding of genetics and almost all had heard of ‘DNA’ (99%; *n* = 422) (Table S2, Supporting Information S1). Most reported knowing what ‘gene’ means (90%; *n* = 385). However, fewer reported knowing the meaning of ‘genome’ (58%; *n* = 250) and ‘sequencing’ (61%; *n* = 263) (Table S3, Supporting Information S1). Self‐perceived understanding of genetics was higher for females (*F*(2) = (4.149), *p* = 0.02) and for those with a degree or higher (*F*(1) = (6.568), *p* = 0.02). Self‐perceived understanding of genetic terms was higher for White/White British respondents than for Asian/Asian British respondents (*F*(5) = (3.008), *p* = 0.01). No other differences across gender, ethnicity, or education were observed.

### 3.2 Objective Knowledge at T1

Overall, respondents (*n* = 427) indicated moderate knowledge of pS at T1 (Table 2): the mean score was 5.69 (SD = 2.59, median = 6.00, range = 0–12), where 0 = low and 12 = high knowledge of pS. There was notable variation in accuracy across items. For instance, for ‘*The prenatal sequencing test looks for all possible gene changes in the baby and parents*’, only 14% (*n* = 61) correctly answered ‘False’. However, more knew that ‘*Prenatal sequencing tests involve reading through the DNA letters in the genome to look for changes*’ was ‘True’ (63%; *n* = 268). Objective knowledge at T1 varied by ethnicity (*F*(5) = (4.609), *p* < 0.05), with higher scores for White/White British respondents compared to those from an Asian/Asian British (*p* = 0.01) and from a mixed ethnic background (*p* = 0.03). Objective knowledge at T1 was positively correlated with self‐rated understanding of genetics (*ρ* = 0.195, *S* = 105,226, *p* < 0.05), understanding of genetic terms (*ρ* = 0.130, *S* = 112,061, *p* = 0.01), and knowledge of genetic terms (*ρ* = 0.431, *S* = 743,097, *p*  < 0.05) at T1, indicating that people's beliefs about their understanding of genetics aligned with what they actually knew.

**TABLE 2:** Number of correct responses to objective knowledge questions at T1 across all respondents.

	Correct response	*N* (%)
Our genome is made up of DNA	True	278 (65)
A change (like a ‘spelling mistake’) in your DNA can cause health problems	True	260 (61)
Most gene changes will affect a person's health	False	88 (21)
Prenatal sequencing tests involve reading through the DNA letters in the genome to look for changes	True	268 (63)
If scans show a baby is not developing as expected, a prenatal sequencing test will always find a genetic change that explains the cause of the problem	False	96 (23)
The prenatal sequencing test looks for all possible gene changes in the baby and parents	False	61 (14)
The prenatal sequencing test compares the DNA from the baby with the DNA from both of the parents	True	261 (61)
It takes 2–3 weeks for parents to receive the results of the prenatal sequencing test	True	213 (50)
If a prenatal sequencing test does not find a diagnosis, the baby definitely won't have a genetic condition that affects their health	False	177 (42)
The test could find a genetic change in the parent's DNA that increases the chance of them having or developing a different health condition	True	266 (63)
The prenatal sequencing test will show whether or not both parents are related to the baby	True	207 (49)
Prenatal sequencing tests could find genetic changes that cannot be understood by doctors and scientists at the present time	True	238 (56)

### 3.3 Self‐Perceived and Objective Knowledge Between T1 and T2

Self‐perceived understanding and knowledge of genetics improved across all respondents post‐intervention (*n* = 427) (Tables S1 and S2, Supporting Information S1). At T2, significantly more respondents described themselves as having ‘Good’ understanding (*p* < 0.05) and more felt they understood the words ‘genome’ (*p* < 0.05) and ‘sequencing’ (*p* < 0.05) compared to T1. Very few reported still not knowing ‘DNA’ (*p* = 0.04).

Objective knowledge scores across all respondents were significantly higher at T2 than at T1 (*V* = 7849, *p* < 0.001) (mean = 8.38, SD = 2.97, median = 9.00, range = 0–12) (Table 3) Significantly higher scores at T2 were also observed across all interventions: leaflet (*V* = 899, *p* < 0.001), animation (*V* = 763, *p* < 0.001) and leaflet plus animation (*V* = 948, *p* < 0.001).

**TABLE 3:** Objective knowledge scores at T1 and T2.

	Total score at T1	Total score at T2	*p*‐value^a^
All groups	*n* = 427	*n* = 427	
Mean	5.69	8.38	< 0.001
95% CI	(5.5–5.9)	(8.1–8.7)	
SD	2.59	2.97	
Range	0–12	0–12	
Q1	4	7	
Median	6	9	
Q3	8	11	
Leaflet	*n* = 130	*n* = 130	
Mean	5.61	7.97	< 0.001
95% CI	(5.2–6.1)	(7.4–8.5)	
SD	2.57	3.11	
Range	0–12	0–12	
Q1	4	6	
Median	6	8	
Q3	7	10.75	
Animation	*n* = 153	*n* = 153	
Mean	5.81	8.61	< 0.001
95% CI	(5.4–6.2)	(8.1–9.1)	
SD	2.55	2.9	
Range	0–11	0–12	
Q1	4	7	
Median	6	9	
Q3	8	11	
Leaflet + animation	*n* = 144	*n* = 144	
Mean	5.65	8.51	< 0.001
95% CI	(5.2–6.1)	(8.0–9.0)	
SD	2.67	2.9	
Range	0–12	0–12	
Q1	4	7	
Median	6	9	
Q3	8	11	

*Note:* When looking at the group overall, total objective scores were significantly higher at T2 than at T1 (*V* = 7849, *p* < 0.001), indicating improvement in knowledge after the individuals experienced the intervention. Assumptions were met for ANCOVA which revealed that, when controlling for total knowledge scores at baseline (T1), the intervention did not significantly contribute to the model. In other words, the increase seen in total objective knowledge scores from T1 to T2 was not driven by the type of intervention to which individuals experienced.

Abbreviations: CI = confidence interval; Q1 = first quartile; Q3 = third quartile; SD = standard deviation.

^a^ Wilcoxon signed rank test.

To compare improvement in objective knowledge scores between interventions, an ANCOVA was performed which allowed us to control for baseline (T1) objective knowledge scores. Two models were run: model 1 included an interaction term between T1 score and Intervention Type, and model 2 included T1 score and Intervention Type as individual terms. The main effect of the T1 score (*F*(1, 426) = 27.21, *p* < 0.001) confirmed that scores at T2 were higher than at T1 across all respondents, indicating that all interventions increased objective knowledge. The main effect of Intervention Type (*F*(2, 426) = 3.16, *p* = 0.043) indicated that T2 scores differed by intervention. However, comparisons between models 1 and 2 revealed a non‐significant interaction between T1 score and Intervention Type (*F*(2, 421) = 2.48, *p* = 0.085), showing no significant difference in the improvement of objective knowledge between interventions.

### 3.4 Respondent Views of pS Information

For each intervention, the majority of respondents reported that the animation (78%; *n* = 119), leaflet (59%; *n* = 77), or animation plus leaflet (72%; *n* = 104), were ‘Quite easy’ or ‘Very easy’ to understand (Table S4, Supporting Information S1). Twenty‐five percent (*n* = 38) of respondents who received the animation reported it was ‘Very easy’ to understand, 9% (*n* = 12) who received the leaflet reported it was ‘Very easy’.

Contradictory to this, respondents (88%–90%) found the information too technical regardless of the intervention type, and just over half reported feeling overwhelmed with information. Most respondents (83%–89%) across the different interventions did however like the way the information was presented and the majority (89%–92%) would have found the information helpful if they had been offered pS (Table 4). Across all respondents, around half (52%) reported that they prefer information presented in video formats, 42% prefer written formats and 6% prefer audio formats (Table S5, Supporting Information S1).

**TABLE 4:** Views on the pS information by intervention type (definitely yes/to some degree).

	*N* (%)		
	Leaflet	Animation	Leaflet + animation
	*n* = 130	*n* = 153	*n* = 145
Did you find the information too technical?	114 (88)	135 (88)	130 (90)
Did you feel overwhelmed with information?	82 (63)	76 (50)	77 (53)
Did you find the information too limited?	72 (55)	72 (47)	84 (58)
Was the explanation about the prenatal sequencing test clear?	42 (32)	38 (25)	42 (29)
Was the information about the possible results clear?	113 (87)	133 (87)	133 (92)
Was the information about what happens after you receive a result clear?	117 (90)	128 (84)	126 (87)
Does the information provide enough detail about where can you find additional information and support?	110 (85)	126 (82)	126 (87)
Did you like the way the information was presented?	108 (83)	136 (89)	128 (88)
Would you have found this information helpful if you were offered a prenatal sequencing test?	120 (92)	136 (89)	133 (92)

Free text comments were left by 118 respondents (27.5%; leaflet: *n* = 33; animation: *n* = 39; leaflet + animation; *n* = 46). Comments specifically about the animation or leaflet were grouped as positive or negative. Positive comments about the animation included that it ‘*helped*’ understanding and that it had ‘*a good voice tone, for a subject that could be emotionally challenging*’. Preferences for video formats generally were also expressed, for example, ‘*video is much better to get our attention*’ and ‘*visual displays are easier to digest*’. Some participants in the animation group highlighted a preference for written formats. Negative comments included that the animation was ‘*very long*’. One comment noted that there was a need for additional details about how the test is performed, and the conditions tested for.

Positive views on the leaflet included that it was ‘*clear and concise*’, ‘*very informative*’ and that it was good to be able to ‘*refer back to it in the future*’. Negative comments included that it was ‘*very technically worded*’ and that it was ‘*too much information to read and take on board*’. Several respondents in the leaflet group expressed a preference for video formats, for example ‘*visual descriptions on video format would have been best*’ Some comments reflected a preference for having both formats to aid consolidation of information.

## 4 Discussion

An animation describing pS was designed with input from parents and clinicians. Our evaluation showed that the animation describing pS was effective in increasing participants' objective knowledge and understanding of pS. The majority of participants who viewed the animation reported that it was easy to understand and that they liked the way the information was presented. The animation, leaflet and animation alongside a leaflet improved knowledge and understanding to a similar extent. Preferences for information format varied, and having multiple formats available was noted by some as beneficial. The animation bridges a gap in available information for parents about pS, where currently there are very few resources that have been developed with parents in mind [10].

Our study found that presenting information about genomic tests through either an animation or written format can improve both objective knowledge and self‐reported understanding. This aligns with previous research on the effectiveness of patient educational resources in enhancing the understanding of genomics more broadly [13, 14, 23] as well as studies specific to the prenatal context. For instance, Stortz and colleagues demonstrated that an educational video on prenatal genetic testing increased patients' knowledge while reducing decisional conflict and regret related to testing options [17]. Similarly, Mulla et al. found that an educational video explaining aneuploidy testing options improved knowledge and self‐reported understanding amongst the women tested [15]. Our findings suggest that an animation about pS can serve as a valuable resource that can be shared with parents alongside the standard written information provided by the NHS GMS, enhancing discussions between parents and professionals.

Notably, while views on the animation and leaflet were generally positive, with most participants reporting that the information was ‘quite easy’ to understand and that they would have found the information helpful if offered pS, the majority of participants also reported that the information was too technical and around half felt that it was overwhelming. In a similar vein, when asked about the different elements of the information, across all interventions, the majority (82%–92%) reported that information about possible results and what happens after results was clear and there was enough detail about additional information and support. However, only around one third felt that the explanation about the technical process of the pS test was clear and around half felt that the information was too limited. More work is needed to understand what additional information is needed to help parents understand the technical aspects of the test. These findings highlight the challenge of providing parents with enough information that they feel informed but not too much detail that they feel overwhelmed. Consideration should be given to creating a second animation that breaks down the technical aspects of pS to a greater extent for those who feel they want more information. Other studies of healthcare animations have successfully used two videos that focus on different elements or steps of the topic area to support incremental learning [22, 27]. Another consideration is that pS is hypothetical to the participants in this study and thus it is possible that their learning or engagement was impacted by their lack of direct personal experience with either invasive testing or pS. A recent meta‐analysis has shown that an animation is more effective when tested with patients than with the general population, possibly because the information is tailored to a particular patient group and setting and because the information is more relevant to the patients so they have greater baseline knowledge and are more engaged with the information [12]. It is also worth noting that the animation and leaflet are intended to complement and not replace discussions with a healthcare professional. In practice, understanding the technical aspects of the test may be less difficult as parents will have already had a conversation with a healthcare professional and the opportunity to seek further clarification. Thus, our findings highlight that a leaflet or animation should complement but not replace a discussion with a healthcare professional so that questions can be addressed in a patient‐centred manner.

We found that preferences for information formats varied between individuals, which is in line with interviews with parents having pS who wanted information available in a range of formats [6]. Offering information about pS in different modalities can support individual learning styles and needs. Non‐native English speakers and those with lower literacy levels could benefit from an animation about pS. This is particularly important as language barriers are known to create inequity in care during pregnancy [28]. Our work, therefore, addresses important issues around equitable access to health information. To broaden the reach of the animation, it has been translated into 11 languages that reflect some of the most common languages spoken in England (Bengali, Urdu, Romanian, Polish, Arabic, Greek, Mandarin, Italian, Portuguese, Spanish, French). Assessing the value of the translated animation in clinical settings to speakers of these languages will be an important next step.

A key strength of this work was the active collaboration with stakeholders during the development of the animation and when undertaking the evaluation. Input from the PPIE Advisory Group, clinicians, and parents at every stage of development helped to ensure that the animation was clinically accurate whilst meeting the needs of parents offered pS. The importance of patients with relevant experiences taking a direct role in the development and evaluation of health interventions has been highlighted [29]. The benefits of embedding PPIE in research from an early stage are increasingly being recognised [30, 31] and PPIE is a valued feature of genomics research in the UK [32]. The work presented here supports these observations. Another strength of this evaluation was the diversity across gender, ethnicity and education level, demonstrating the generalisability of the findings.

## 5 Limitations

A limitation of our evaluation was that we tested knowledge immediately following information delivery which is unlikely to represent the real‐world application of the interventions. Examining the impact of interventions on the improvement of longer‐term knowledge would be valuable. In addition, we did not include parents offered pS whose needs may differ from those of the parents in this study. This is an important consideration as parents offered pS in a clinical setting are often asked to make decisions about testing under stressful conditions [4, 5, 6, 7]. Future evaluation with parents offered pS clinically is important. Amongst the 36 respondents who stated speaking a main language different from English, 22 different languages were reported; this combination of small sample size and wide linguistic variation means that we were unable to control for the possible effect of participants' proficiency in English on their knowledge acquisition—a further limitation to this work.

## 6 Conclusion

An animation about pS, developed with input from a range of stakeholders, can improve knowledge about pS. The animation has been made freely available online and has been translated into multiple languages. Future research should include a formal evaluation of the animation in the clinical setting and explore the value of the translated versions for parents who speak those languages. Exploration of appropriate information formats and resources for parents with learning disabilities is also needed.

## Ethics Statement

The evaluation was approved by the Health Research Authority (HRA) and the East of Scotland Research Ethics Service REC 1 (21/ES/0073).

## Consent

All participants were over the age of 18. Consent was obtained from all participants to take part in this study and for this information to be published.

## Conflicts of Interest

The authors declare no conflicts of interest.

## Supporting information

Supporting Information S1

Tables S1–S5

## Data Availability

The data that support the findings of this study are available from the corresponding author upon reasonable request.
