# Supplementary material for: Can an Animation Improve Parents' Knowledge and How Does It Compare to Written Information? Development and Survey Evaluation of an Animation for Parents About Prenatal Sequencing
Source: Prenat Diagn. 2025 Apr 2;46(5-6):737–45. doi: 10.1002/pd.6792 (PMC13170059; doi:10.1002/pd.6792)
Supplement: Supplementary file 1 — Supporting Information S1 [file PD-46-737-s002.docx]

# **Supplementary Information**

**Development of the prenatal sequencing animation**

The prenatal sequencing (pS) animation was developed by the EXPRESS research team with input from: a) members of the EXPRESS Public and Patient Involvement (PPI) group (made up of patient charity representatives, and a bereaved parent and academic), b) parents who had been offered testing in pregnancy, and c) clinicians, including genetic counsellors, clinical geneticists, clinical scientists, fetal medicine consultants and fetal medicine midwives. The animation was developed over three main phases: 1) Content and script development, 2) Script and storyboard development, and 3) Animation development.

**Phase 1: Content and script development**

1. *Content development: Research team*

At this first stage in January 2022, the research team gathered together information from a range of sources that would help inform the content of the animation. Information was sourced from: a) the parent information leaflets and consent forms used in the NHS Genomic Medicine Service pS service^[[1]](#endnote-1)^, b) professional guidance on pre-test counselling content for pS^[[2]](#endnote-2),^^[[3]](#endnote-3),^^[[4]](#endnote-4)^, c) the DISCERN Genetics tool, which is used to assess the quality of patient health information^[[5]](#endnote-5)^, and d) findings from qualitative interviews with parents offered pS^[[6]](#endnote-6)^. The research team collated this information with the view to gathering feedback from the PPI group and clinicians about the suggested content of the animation.

1. *Content development: PPI group and clinicians*

The PPI group were in full support of the idea of developing an animation about pS and raised important points such as the need to refrain from using overly complex, scientific language in the animation and to avoid an animation that was too long. The clinicians were also positive about the development of an animation, feeling that it would be a useful aid for their clinic discussions. They provided helpful suggestions regarding the content such as the importance of balancing accurate information without overwhelming the viewer.

1. *First draft of script developed*

In February 2022, the research team produced a draft script (version 1) to accompany the animation. The script was guided by the professional guidance documents described above^[[7]](#endnote-7),^^[[8]](#endnote-8),^^[[9]](#endnote-9)^, the feedback from qualitative interviews with parents offered pS^[[10]](#endnote-10)^, and the suggestions made by the PPI group and clinicians.

**Phase 2: Script and storyboard development**

1. *Orinoco employed to develop the animation*

In March 2022, the research team employed Orinoco - a digital communications and content creation agency, to produce the animation. In May 2022, Orinoco invited the research team, clinicians, and PPI group members to a 90-minute ‘discovery session’. This session provided the opportunity to establish the tone and ‘visual language’ for the animation.


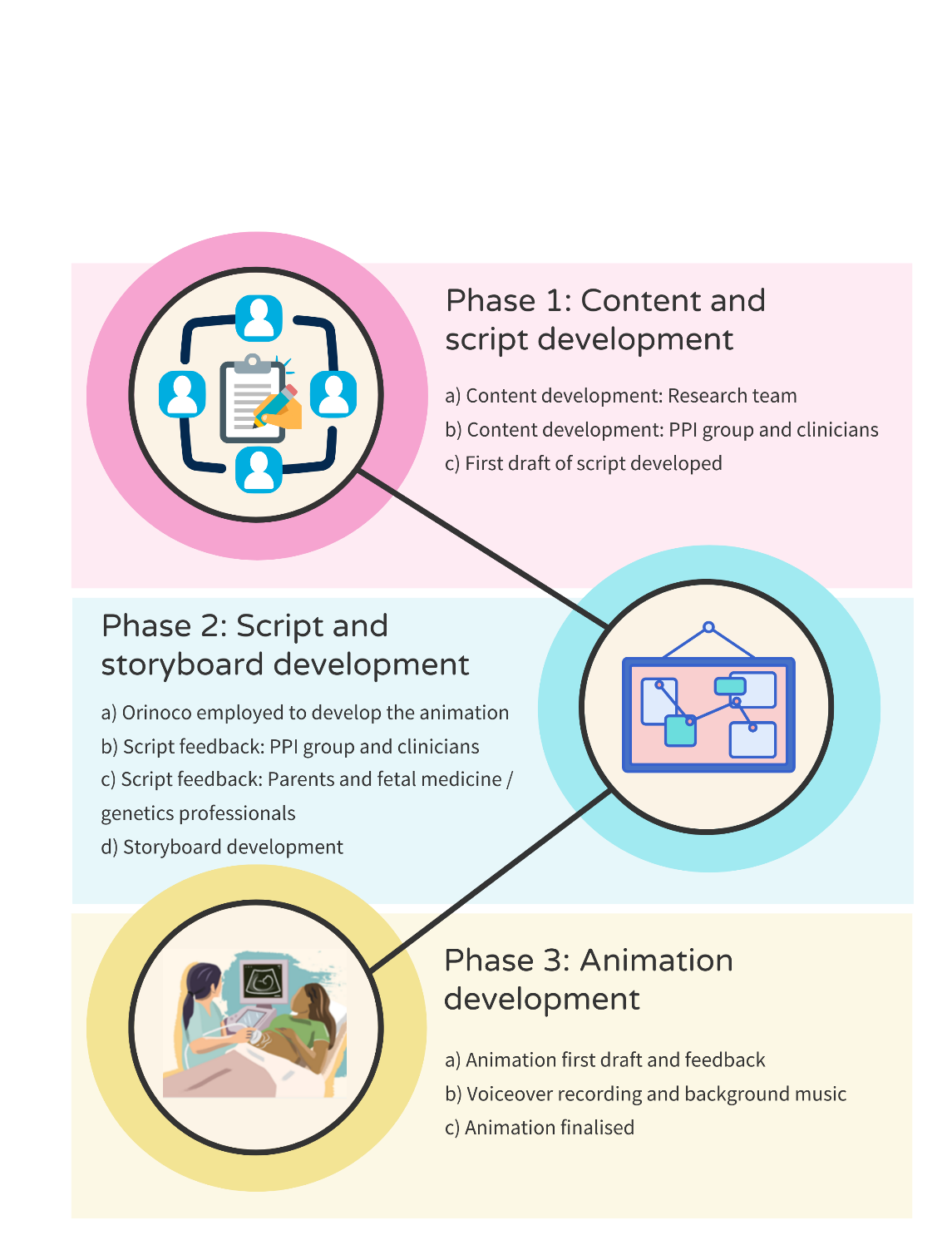


1. *Script feedback: PPI group and clinicians*

Following the discovery session, Orinoco made edits to the draft script and returned it for feedback. The PPI group felt that a priority for parents would be detailed information about the range of possible results from pS and suggested that this section of the script be improved to include more detail. With help from EXPRESS clinicians, a more informative draft script (version 2) was developed.

1. *Script feedback: Parents and fetal medicine and genetics professionals*

In August 2022, the script (version 2) was circulated for final feedback to parents who had offered to take part in the EXPRESS study, and to fetal medicine and genetics health professionals across England who were involved in pS service delivery. Parents reported liking the analogy used in the script of the genome as the body’s ‘instruction manual’ and felt the script was easy to understand. Clinicians were also positive but had some suggestions for improvement. These suggestions included clarification that not everyone will receive a diagnosis and being explicit that a ‘no findings’ result from pS does not mean the baby does not have a genetic change.

1. *Storyboard development*

In parallel to awaiting script feedback from the various collaborators, Orinoco began development of the imagery and storyboard for the animation. A draft storyboard was shared with the PPI group and clinicians in November 2022. Both groups agreed on the warm colour palette and animation style that had been used, but highlighted the need to include couples from different ethnic and cultural backgrounds as well as couples with different sexual orientations. Further suggestions from the clinicians included swapping some depictions of laboratory and scientific equipment for ones actually used in prenatal sequencing. Members of the PPI group were also uncomfortable with a scene depicting a needle, which could be frightening to some, and another scene in which a parent was cradling a baby, viewing this as insensitive for a parent who may be considering ending a pregnancy or whose baby could have a lethal or life-limiting condition.

**Phase 3: Animation development**

1. *Animation first draft and feedback*

By January 2023, changes to the storyboard were complete. Orinoco shared an ‘animatic’ (a preliminary version of the animation with examples of transitions between scenes) with the research team, PPI group and clinicians for feedback on the transitions and animation style. All agreed that the transitions between scenes required speeding up, but were happy with the overall feel of the animation.

1. *Voiceover recording and background music*

Based on suggestions from the PPI group that the narration use a warm, female voice, Orinoco drew up a shortlist of voiceover professionals. The team selected the voice they felt had the best warmth of tone. The voiceover was recorded in a ‘virtual studio’ in March 2023. A research team member sat in on the recording session to help direct the voiceover professional and answer any questions about the appropriate tone and flow at various points throughout the recording. Finally, Orinoco selected a shortlist of background tracks and the team selected the one they felt was the most calm and least distracting for those watching the animation.

1. *Animation finalised*

In March 2023, Orinoco delivered the final animation in two versions, one with English subtitles and one without.

1. NHS England and NHS Improvement. Guidance document: Rapid exome sequencing service for fetal anomalies testing [internet]. Available from: https://norththamesgenomics.nhs.uk/wp-content/uploads/2023/08/Rapid-Exome-Sequencing-Guidance.pdf. [↑](#endnote-ref-1)
2. Joint position statement from the international society for prenatal diagnosis (ISPD), the society for maternal fetal medicine (SMFM), and the perinatal quality foundation (PQF) on the use of genome‐wide sequencing for fetal diagnosis. *Prenat Diagn*. 2018;38(1):6‐9. [↑](#endnote-ref-2)
3. Abou Tayoun AN, Spinner NB, Rehm HL, Green RC, Bianchi DW. Prenatal DNA sequencing: clinical, counseling, and diagnostic laboratory considerations. *Prenat Diagn*. 2018;38(1):26‐32. [↑](#endnote-ref-3)
4. Monaghan KG, Leach NT, Pekarek D, Prasad P, Rose NC, Rose NC. The use of fetal exome sequencing in prenatal diagnosis: a points to consider document of the American College of Medical Genetics and Genomics (ACMG). *Genet Med*. 2020;22(4):675‐680. [↑](#endnote-ref-4)
5. Shepperd S, Farndon P, Grainge V, et al. DISCERN-Genetics: quality criteria for information on genetic testing. *Eur J Hum Genet*. 2006; 14(11): 1179-1188. [↑](#endnote-ref-5)
6. McInnes-Dean H, Mellis R, Daniel M, Walton H, Baple EL, Bertoli M, et al. 'Something that helped the whole picture': Experiences of parents offered rapid prenatal exome sequencing in routine clinical care in the English National Health Service. Prenat Diagn. 2024 Apr;44(4):465-479. doi: 10.1002/pd.6537. Epub 2024 Mar 5. PMID: 38441167. [↑](#endnote-ref-6)
7. Joint position statement from the international society for prenatal diagnosis (ISPD), the society for maternal fetal medicine (SMFM), and the perinatal quality foundation (PQF) on the use of genome‐wide sequencing for fetal diagnosis. *Prenat Diagn*. 2018;38(1):6‐9. [↑](#endnote-ref-7)
8. Abou Tayoun AN, Spinner NB, Rehm HL, Green RC, Bianchi DW. Prenatal DNA sequencing: clinical, counseling, and diagnostic laboratory considerations. *Prenat Diagn*. 2018;38(1):26‐32. [↑](#endnote-ref-8)
9. Monaghan KG, Leach NT, Pekarek D, Prasad P, Rose NC, Rose NC. The use of fetal exome sequencing in prenatal diagnosis: a points to consider document of the American College of Medical Genetics and Genomics (ACMG). *Genet Med*. 2020;22(4):675‐680. [↑](#endnote-ref-9)
10. McInnes-Dean H, Mellis R, Daniel M, Walton H, Baple EL, Bertoli M, et al. 'Something that helped the whole picture': Experiences of parents offered rapid prenatal exome sequencing in routine clinical care in the English National Health Service. Prenat Diagn. 2024 Apr;44(4):465-479. doi: 10.1002/pd.6537. Epub 2024 Mar 5. PMID: 38441167. [↑](#endnote-ref-10)
