# Supplementary material for: Can an Animation Improve Parents' Knowledge and How Does It Compare to Written Information? Development and Survey Evaluation of an Animation for Parents About Prenatal Sequencing
Source: Prenat Diagn. 2025 Apr 2;46(5-6):737–45. doi: 10.1002/pd.6792 (PMC13170059; doi:10.1002/pd.6792)
Supplement: Supplementary file 2 — Tables S1–S5 [file PD-46-737-s001.docx]

# **Supporting Information**

**Table S1.** Participants who reported a main language spoken that was not English.

| **Asian or Asian British** | **16** |
| --- | --- |
| Arabic | 1 |
| Cantonese | 2 |
| Hindi | 2 |
| Italian | 1 |
| Malayalam | 1 |
| Marathi | 1 |
| Nepali | 1 |
| Pashto | 1 |
| Portuguese | 1 |
| Punjabi | 1 |
| Urdu | 4 |
| **Black or Black British** | **9** |
| Arabic | 1 |
| French | 1 |
| Hausa | 1 |
| Igbo | 1 |
| Luganda | 2 |
| Shona | 2 |
| Yoruba | 1 |
| **Mixed ethnicity** | **1** |
| Spanish | 1 |
| **Other** | **1** |
| Arabic | 1 |
| **Prefer not to say** | **1** |
| Farsi | 1 |
| **White or White British** | **8** |
| Bulgarian | 1 |
| Italian | 1 |
| Polish | 2 |
| Portuguese | 1 |
| Russian | 1 |
| Spanish | 2 |
| **Grand Total** | **36** |

| **Table S2.** Perceived understanding of genetics and genetics terms across all respondents. | | |
| --- | --- | --- |
|  | **N%** | |
|  | **T1** | **T2** |
| *How would you describe your understanding of genetics?* |  |  |
| None | 92 (22) | 12 (3) |
| Some | 297 (70) | 226 (53) |
| Good | 34 (8) | 189 (44) |
|  |  |  |
| *Have you heard of these words before…* |  |  |
| DNA |  |  |
| Yes | 422 (99) | 423 (99) |
| No | 4 (1) | 4 (1) |
| Gene |  |  |
| Yes | 297 (70) | 409 (70) |
| No | 92 (22) | 18 (22) |
| Genome |  |  |
| Yes | 291 (68) | 363 (85) |
| No | 135 (32) | 64 (15) |
| Sequencing |  |  |
| Yes | 307 (72) | 361 (85) |
| No | 119 (28) | 66 (15) |
|  |  |  |

| **Table S3.** Perceived knowledge of genetics across all respondents**.** | |  |
| --- | --- | --- |
|  | **N (%) Strongly/Agree** | |
|  | **T1** | **T2** |
|  |  |  |
| Do you know what these words mean? |  |  |
| DNA | 395 (92) | 376 (88) |
| Gene | 385 (90) | 371 (87) |
| Genome | 250 (58) | 311 (73) |
| Sequencing | 263 (61) | 322 (75) |
|  |  |  |
|  |  |  |

**Table S4.** Ease of understanding of the prenatal sequencing information by Intervention type.

|  | **N (%)** | | |
| --- | --- | --- | --- |
|  | **Leaflet** | **Animation** | **Leaflet + Animation** |
|  | n = 130 | n = 152 | n = 115 |
| *Was the information easy to understand?* |  |  |  |
| Very easy | 12 (9) | 38 (25) | 35 (24) |
| Quite easy | 65 (50) | 81 (53) | 69 (48) |
| Quite hard | 45 (35) | 27 (18) | 38 (26) |
| Very hard | 8 (6) | 6 (4) | 3 (2) |
|  |  |  |  |

**Table S5.** Preferred format for prenatal sequencing information.

|  | **N (%)** | | | |
| --- | --- | --- | --- | --- |
|  | **All groups** | **Leaflet** | **Animation** | **Leaflet + Animation** |
|  | n = 428 | n = 131 | n = 132 | n = 133 |
|  |  |  |  |  |
| Video | 224 (52) | 58 (45) | 91 (59) | 75 (52) |
| Written | 179 (42) | 63 (48) | 53 (35) | 63 (43) |
| Audio | 25 (6) | 9 (7) | 9 (6) | 7 (5) |
|  |  |  |  |  |
